# *Erhaia* Davis & Kuo (Gastropoda, Rissooidea, Amnicolidae) also in Bhutan

**DOI:** 10.3897/zookeys.679.13326

**Published:** 2017-06-08

**Authors:** Edmund Gittenberger, Sherub Sherub, Björn Stelbrink

**Affiliations:** 1 Naturalis Biodiversity Center, P.O. Box 9517, NL-2300RA Leiden, The Netherlends; 2 Ugyen Wangchuck Institute for Conservation and Environment, Bumthang, Bhutan; 3 Department of Animal Ecology & Systematics, Justus Liebig University, Heinrich-Buff-Ring 26-32 IFZ, D-35392 Giessen, Germany

**Keywords:** *Erhaia*, *Akiyoshia*, 16S, taxonomy, distribution, Nepal, Bhutan

## Abstract

The occurrence of at least one species of *Erhaia* in Bhutan, viz. *Erhaia wangchuki* **sp. n.**, is confirmed by DNA sequencing. A second unnamed species from Bhutan, that might be congeneric, is known from only a single shell. According to the molecular analysis, *E. wangchuki* is most closely related to a still undescribed *Erhaia* species from China. These two species together with *E. jianouensis* and *Akiyoshia kobayashii*, both also from China, form a well supported clade. Awaiting additional molecular data, the apparent inconsistency regarding *Erhaia* versus *Akiyoshia* is not dealt with here. The extant true sister species of *E. wangchuki* could be among the four SE Himalayan species from Bhutan and Nepal that are classified with *Erhaia* on the basis of conchological data only.

## Introduction

The extremely speciose superfamily Rissooidea, with over 400 recent genera ‘one of the largest gastropod families’ (Wilke et al. 2001: 1), encompasses very many species that cannot be identified on the basis of only shell characters, distribution and ecology. Anatomical characters may additionally be used, when the equipment for dissection is available, but even that technically demanding approach does not always bring conclusive results. Therefore, molecular analyses are advisable in cases like this. This has resulted in a more reliable classification allowing these snails to be used as ecological indicator species on the one hand and model organisms in historical biogeography on the other hand.

After its description by Davis and Kuo in Davis et al. (1985), the genus *Erhaia* turned out to be widespread in Asia. From Nepal, two species of *Erhaia* are reported by Nesemann et al. (2007) on the basis of shell shape and ecology. Here, we report the occurrence of a species of *Erhaia* in Bhutan, confirmed by a molecular analysis. The shell of a species that might be congeneric is additionally described but without naming it.

## Material and methods

**Figure 1.**
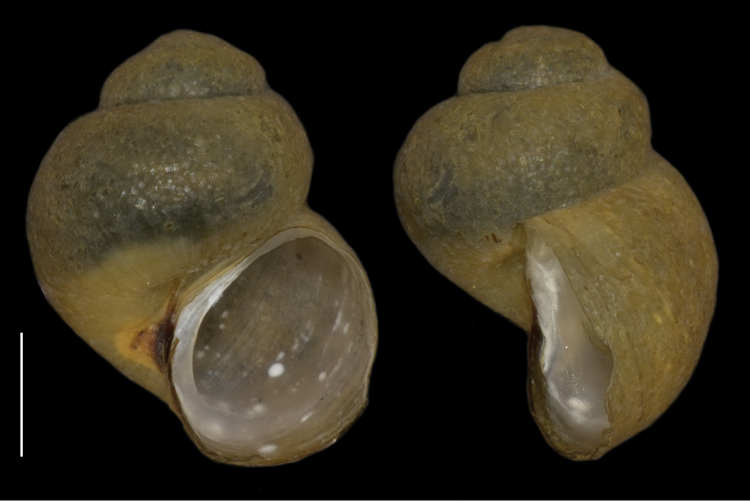
*Erhaia wangchuki* sp. n., sequenced paratype; scale bar 0.5 mm (photographs by B.S.); Bhutan, district Wangdue Phodrang, Gangchhu, 2883 m alt.; 27°26'N, 90°11'E; Jigme Wangchuk leg. 21.iii.2015.

**Figure 2.**
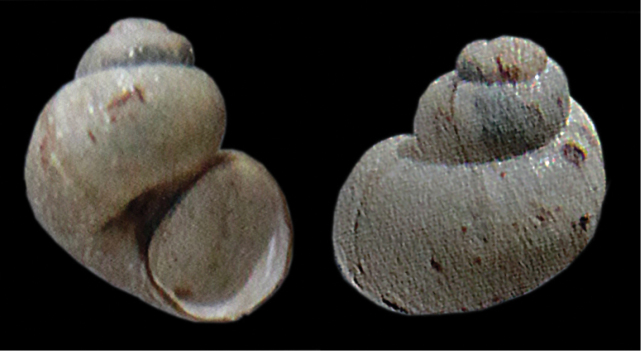
*Erhaia wangchuki* sp. n., holotype NBCB1013, measurements 2.15×1.77 mm (photographs by E.G.); Bhutan, district Wangdue Phodrang, Gangchhu, 2883 m alt.; 27°26'N 90°11'E; Jigme Wangchuk leg. 21.iii.2015.

The first author coincidentally collected a minute snail in a marshy source area in Bhutan, in the district Thimphu, W of Geneykha at 2825 m altitude. The locality could not yet be visited again. The specimen got lost after being photographed with a Ricoh WG-4 digital camera, using the extreme macro facility. Some equally small snails were found by Jigme Wangchuk of the Ugyen Wangchuck Institute for Conservation and Environment, Bumthang, in a source in the district Wangdue Phodrang at 2883 m altitude, and preserved in alcohol 70%. One specimen (Fig. 1) of the latter sample, a paratype, was photographed with a digital microscope system (KEYENCE VHX-2000; KEYENCE Corp., Itasca, IL, USA) and then used for a molecular analysis. Another shell, the holotype (Fig. 2), was photographed with a Canon EOS 7D, using a Canon extension tube EF25 and a Sigma DC 18-50 mm lens.

DNA was isolated using standard protocols for molluscs (see Stelbrink et al. 2016 for details). A mitochondrial DNA fragment (16S rRNA) was amplified and sequenced (GenBank accession number: KY798003). In addition, a maximum likelihood tree (Fig. 4) was obtained using RAxML BlackBox (substitution model: GTR+Γ, 100 bootstrap replicates; Stamatakis et al. 2008) by using the 16S rRNA dataset of Liu et al. (2014).

## Results

Our molecular analysis resulted in a tree (Fig. 4) with *Erhaia wangchuki* sp. n. as the sister taxon of an undescribed *Erhaia* sp. from China, Guangxi, Xiangjiang river at Quanzhou (after Liu et al., 2014: 22). The sister taxon of these two species is unclear, but a clade formed by four species, viz. *E. wangchuki*, *Erhaia* spec., *Akiyoshia kobayashii* Kuroda & Habe, 1958 and *E. jianouensis* (Liu & Zhang, 1979), is highly supported.

## Systematics

### Superfamilia Rissooidea Gray, 1847

#### Family Amnicolidae Tryon, 1863

##### Genus Erhaia

Taxon classificationAnimaliaGastropodaAmnicolidae

Davis & Kuo, 1985

###### Type species.

*Erhaia daliensis* Davis & Kuo, in Davis, Kuo, Hoagland, Chen, Yang and Chen, 1985.

##### Erhaia wangchuki sp. n.

Taxon classificationAnimaliaGastropodaAmnicolidae

http://zoobank.org/304DE8F4-959A-4C7E-A497-00DA959CB99D

Figs 1 , 2

###### Material.

District Wangdue Phodrang, Gangchhu (Figs 5, 6), 2883 m alt.; 27°26'N 90°11'E; Jigme Wangchuk leg. 21.iii.2015. National Biodiversity Centre, Serbithang, Thimphu [holotype NBCB1013, paratypes NBCB1014/2].

###### Shell.

Conical, broader than high, with a flat apex because the initial ¾-1 whorl is planispiral; 3¼ whorls in total. Body whorl large, the height of the aperture exceeds that of the spire. Aperture with a broadly rounded outer lip and a nearly straight parietal side, so that a columellar border is hardly recognizable. Growthlines moderately strong, with a more prominent periostracal ridge at more or less regular distances. Teleoconch whorls broadly shouldered and separated by a deeply incised suture. Aperture oblique ovoid, smooth inside; apertural edge not touching the penultimate whorl. Umbilicus widely open. The holotype is the largest shell and measures 2.15×1.77 mm.

The shell differs from the shells of the three ‘*Erhaia*’ species reported from Nepal by Nesemann et al. (2007) by the large body whorl, the relative height of the aperture, and by being broader than high. The other species that are referred to as *Erhaia* in the literature, from areas that are further apart than Bhutan and Nepal, also have different combinations of character states.

###### Notes.

This species is known from the source of the Gangzetem brooklet, emerging from an underground spring aquifer surrounded by blue pine (*Pinus wallichina*) and a small open meadow (Figs 5, 6). The stream bed substrate, viz. pebbles, small rocks and parts of plants, is covered with dark-green algae, housing an abundant diversity of aquatic invertebrates. Alongside the brooklet are rhododendrons (*Rhododendron thomsonii*, *R. arboretum*, *R. kesangae*), berries (*Berberies asiatica*, *Rosa sericea*), betula (*Betula utilis*), larch (*Larix griffithii*), daphne (*Daphne bholua)* and remnants of dead dwarf bamboo (*Yushania microphyllus*).

A farm road to the villages of Gangphel and Zizi crosses over the stream. The source is very close (~50m) to that road. The stream also spins a *chhukhor*, i.e. a water powered prayer wheel. At the very outlet of the stream is a water tank, which supplies drinking water to Damchu Lhakhang. The brooklet meanders into the Phobji main stream, and measures about 1100 meters. During the pre-monsoon (21.03.2015) and post-monsoon (29.11.2015), physiochemical properties of the stream were measured. The water is almost neutral (pH 7.06, 7.58) and has a nearly stable temperature (6.76, 6.20^º^C).

###### Etymology.

*wangchuki*, after Jigme Wangchuk, who discovered these minute snails.

##### Erhaia

Taxon classificationAnimaliaGastropoda Amnicolidae

sp.

Fig. 3

###### Material.

District Thimphu, 4.5 km E of Chuzom, W of Genekha, 2750 m alt.; 27°19'N 89°36'E; E. Gittenberger leg. 21.vi.2012.

###### Shell.

Elongated ovoid, higher than broad, with a last whorl measuring more than ¾ of the total shell height; aperture attached to the penultimate whorl for less than ⅓ of the parietal-columellar side. Umbilicus very narrow. Shell height ca. 2 mm.

###### Notes.

The shell is most similar in size and shape to ‘*Erhaia’ chandeshwariensis* Nesemann & Sharma, 2007, and ‘*Erhaia’ banepaensis* Nesemann & Sharma, 2007, as figured by Nesemann et al. (2007: 78, figs 4–5). *Erhaia wangchuki* sp. n. differs clearly by the broader shell with a lower spire.

**Figure 3.**
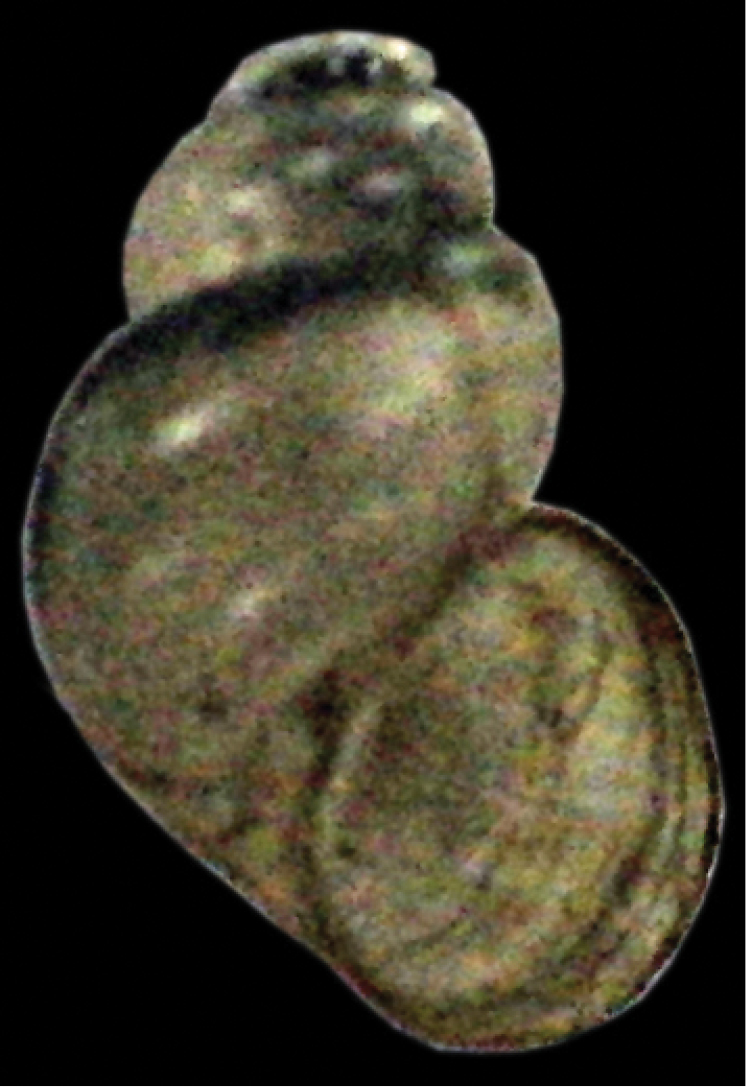
*Erhaia* spec., measurements c. 2.0×1.35 mm (photograph by E.G.). Bhutan, district Thimphu, 4.5 km E of Chuzom, W of Genekha, 2750 m alt.; 27°19'N 89°36'E; E. Gittenberger leg. 21.vi.2012.

**Figure 4.**
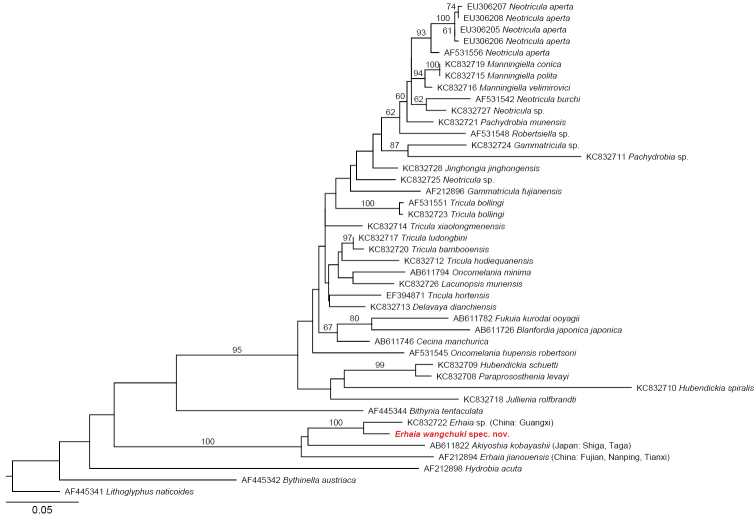
Maximum likelihood tree based on the 16S rRNA dataset of Liu et al. (2014). Numbers on branches denote bootstrap values >50.

**Figures 5–7.**
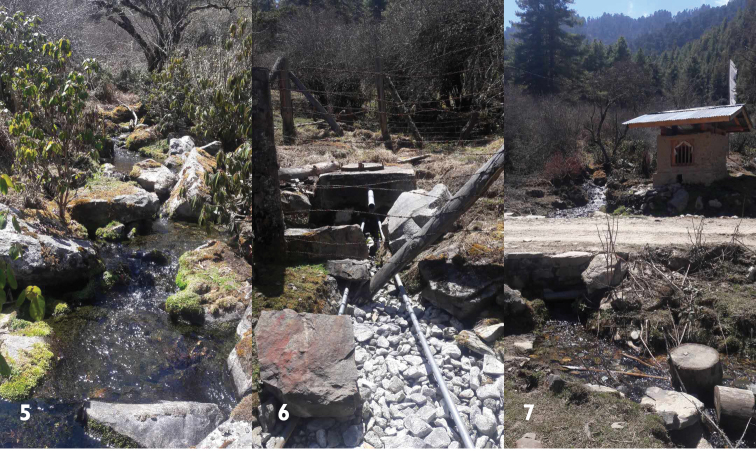
The Gangzetem brooklet (**5**), with the watertank at the source (**6**), and the site where the brooklet crosses the road (**7**). Photographs by Damber Bdr Chhetri.

## Discussion

After its introduction by Davis et al. (1985), the classification of the genus *Erhaia* remained uncertain for some time. Wilke et al. (2000) eventually published maximum likelihood phylogenies based on sequences of both nuclear and mitochondrial markers, viz. 18S, and 16S and COI, and a combination of all three markers. COI supported a clade formed by *Erhaia* and the combined *Amnicola* Gould & Haldeman, 1840 and *Bythinella* Moquin-Tandon, 1856. Both 18S and 16S placed *Amnicola* next to the combined *Erhaia* and *Moria* Kuroda & Habe, 1958. The combined data showed the combined *Erhaia* and *Moria* as the sister group of *Amnicola*, and these three genera together in a sister group relation with *Bythinella*. Later on, maximum likelihood phylogenies were published using data from 18S and COI and a combination of both (Wilke et al. 2001). According to this study, *Erhaia*, *Amnicola* and *Marstoniopsis* Van Regteren Altena, 1936 together form a clade in the COI and in the combined tree as the sister group of *Bithynella*; the same three genera cluster together in the 18S-based tree, where the position of *Bythinella* remains unresolved. Liu et al. (2014), while including other genera in their molecular study, once again confirmed the systematic position of *Erhaia* together with *Akiyoshia* Kuroda & Habe, 1954 and *Bythinella* within the Amnicolidae.

*Erhaia* is known by several species in China, from the Yangtze River drainage and, by one species, from the Mekong River drainage (Davis and Kang 1995, Davis and Rao 1997). The probability that *Erhaia* is represented in Nepal indeed (Nesemann et al. 2007) is considerably enhanced by the anatomically confirmed occurrence of *E. nainitalensis* Davis & Rao, 1997, in Nainital in northern India west of Nepal, and *E. wangchuki* sp. n. in Bhutan east of Nepal. It may be hypothesized that there is a radiation in *Erhaia* along the southern border of the Himalaya, far west and northwest of its large range in China. With hardly any spring area in Bhutan searched for micro-snails, additional species may wait for discovery there at least.

## Supplementary Material

XML Treatment for Erhaia

XML Treatment for Erhaia wangchuki

XML Treatment for Erhaia
